# Folate-Equipped Cationic Liposomes Deliver Anti-MDR1-siRNA to the Tumor and Increase the Efficiency of Chemotherapy

**DOI:** 10.3390/pharmaceutics13081252

**Published:** 2021-08-13

**Authors:** Daniil V. Gladkikh, Aleksandra V. Sen′kova, Ivan V. Chernikov, Tatyana O. Kabilova, Nelly A. Popova, Valery P. Nikolin, Elena V. Shmendel, Mikhail A. Maslov, Valentin V. Vlassov, Marina A. Zenkova, Elena L. Chernolovskaya

**Affiliations:** 1 Institute of Chemical Biology and Fundamental Medicine, Siberian Branch of the Russian Academy of Sciences, 8 Lavrentiev Avenue, 630090 Novosibirsk, Russia; medulla35@gmail.com (D.V.G.); alsenko@mail.ru (A.V.S.); chernikovivanv@gmail.com (I.V.C.); kabilova@niboch.nsc.ru (T.O.K.); vvlassov@mail.ru (V.V.V.); marzen@niboch.nsc.ru (M.A.Z.); 2 The Federal Research Center Institute of Cytology and Genetics, Siberian Branch of the Russian Academy of Sciences, 10 Acad. Lavrentjev Avenue, 630090 Novosibirsk, Russia; nelly@bionet.nsc.ru (N.A.P.); nikolin@bionet.nsc.ru (V.P.N.); 3 Institute of Fine Chemical Technologies, MIREA, Russian Technological University, 119571 Moscow, Russia; shmelka_87@mail.ru (E.V.S.); mamaslov@mail.ru (M.A.M.)

**Keywords:** cationic liposomes, folate-containing lipoconjugate, folic acid, transfection, targeted delivery, siRNA, *MDR1* gene, xenograft tumor model

## Abstract

In this study, we examined the in vivo toxicity of the liposomes F consisting of 1,26-bis(cholest-5-en-3-yloxycarbonylamino)-7,11,16,20-tetraazahexacosan tetrahydrochloride, lipid-helper 1,2-dioleoyl-sn-glycero-3-phosphoethanolamine and folate lipoconjugate (*O*-{2-[*rac*-2,3-di(tetradecyloxy)prop-1-yloxycarbonyl]aminoethyl}-*O*’-[2-(pteroyl-L-glutam-5-yl)aminoethyl]octadecaethyleneglycol) and investigated the antitumor effect of combined antitumor therapy consisting of MDR1-targeted siMDR/F complexes and conventional polychemotherapy using tumor xenograft initiated in immunodeficient mice. Detailed analysis of acute and chronic toxicity of this liposomal formulation in healthy C57BL/6J mice demonstrated that formulation F and parent formulation L (without folate lipoconjugate) have no acute and chronic toxicity in mice. The study of the biodistribution of siMDR/F lipoplexes in SCID mice with xenograft tumors formed by tumor cells differing in the expression level of folate receptors showed that the accumulation in various types of tumors strongly depends on the abandons of folate receptors in tumor cells and effective accumulation occurs only in tumors formed by cells with the highest FR levels. Investigating the effects of combined therapy including anti-MDR1 siRNA/F complexes and polychemotherapy on a multidrug-resistant KB-8-5 tumor xenograft in SCID mice demonstrated that siMDR/F increases the efficiency of polychemotherapy: the treatment leads to pronounced inhibition of tumor growth, reduced necrosis and inflammation, and stimulates apoptosis in KB-8-5 tumor tissue. At the same time, it does not induce liver toxicity in tumor-bearing mice. These data confirm that folate-containing liposome F mediated the extremely efficient delivery of siRNA in FR-expressing tumors in vivo and ensured the safety and effectiveness of its action.

## 1. Introduction

In the last few decades, there has been an increasing interest in the application of therapeutic nucleic acids (NA, either small interfering RNA [siRNA], antisense oligonucleotides or immunostimulating NA) in the treatment of hereditary and acquired diseases [1,2,3]. The effectiveness of the therapeutic action of NA-based drugs is determined by three main components: the choice of a target to be affected to prevent or to reverse the development of a pathology, the design of an effective NA that effectively acts on the target at the lowest possible concentration, and the development of a method for delivering this NA to the target cells. In many cases, the effective bio-performance of NA-based therapeutics in vivo has been provided by a delivery vehicle [4]. Thus, effective and targeted delivery of therapeutic NA is the main issue determining their biological activity, and compliance of the carrier structure with numerous parameters is required to create effective delivery systems.

Compositions containing cationic lipids, which effectively interact with negatively charged NA to form a lipoplex, are widely used for NA delivery. It has been shown that cationic lipoplexes predominantly accumulate in highly vascularized organs—the liver, kidneys, heart, and lungs—but they do not have pronounced specificity. Therefore, lipoplexes that carry ligands to certain cellular receptors have been used in the development of NA delivery vehicles [5,6] to reduce the overall toxic effect on the body and to ensure the accumulation of an effective therapeutic dose (TD) of NA in the target organ. The attachment of ligands such as transferrin, folate, carbohydrates, growth factors, antibodies, and low-density lipoprotein (LDL) receptor ligands to lipoplexes has been successfully applied for selective delivery of NA to target various cells [7,8,9,10,11,12].

Cationic liposomes exhibiting high efficacy in the delivery of NA in cell culture are less effective in the body due to pronounced toxicity associated with their positive charge, interaction with proteins and blood cells, and elimination by the reticuloendothelial system (RES), mainly by macrophages in the systemic circulation and Kupffer cells in the liver [13,14]. Therefore, the main issues of creating NA-based therapeutic formulations are stabilizing NA in the bloodstream, ensuring its preferential accumulation in the target cells, reducing the rate of NA elimination, and improving the bioavailability and safety.

Intravenous (IV) administration is commonly used in the clinic because it leads to immediate exposure of all organs to the drug, but it may cause undesired side effects, including toxicity. After getting into the bloodstream, the accumulation of NA in cells and organs is provided both by the properties of the vasculature of the organ, which determine the retardation of NA in the organ and the access of NA-containing complexes directly to the cell membrane, and by the properties of the complexes themselves and their ability to bind to the membrane and be internalized by the cells. With this administration route, the use of targeted delivery systems, equipped with ligands for receptors specifically expressed on the surface of the target cells, is preferable due to the increased selectivity and, consequently, decreased potential systemic toxicity. The delivery systems, which are capable of specifically transferring NA to particular cells or tissue components and of avoiding delivery into untargeted healthy cells, have important advantages upon delivery in vivo. The overexpression of the folate receptor (FR) has been observed on the surface of various epithelial cancer cells, including cancers of the ovary, uterus, lung, kidney, breast, colon, prostate, and brain [15,16,17], making folate a useful ligand for tumor targeting [18]. Adding the folate ligand to delivery systems may lead to enhanced potency due to increased deposition in tumors as well as increased cellular uptake. Some authors have reported in vivo studies with folate-modified delivery systems including liposomes [19,20,21,22] lipid- and polymer-based nanoparticles [23,24]. They have shown an effective accumulation of siRNA or plasmid DNA (pDNA) in tumor xenograft of immunodeficient mice after systemic administration, followed by the therapeutic change of target gene expression in tumors and, as a result, antitumor activity. Several studies have reported no or mild toxic effects on vital organs and blood biochemistry after IV administration of siRNA, when FR-targeted liposomes [25] or polycationic oligomer-based nanoparticles were used as the delivery agent [26,27]. An approach involving the use of FR-targeted liposomes has also shown its effectiveness when their complexes with pDNA were administered intraperitoneally [28,29]. The authors reported no significant toxic effects on body weight, blood biochemistry, and internal organs.

We previously developed an effective liposomal NA delivery system based on the polycationic amphiphile 1,26-bis(cholest-5-en-3-yloxycarbonylamino)-7,11,16,20-tetraazahexacosan tetrahydrochloride (2X3) and the helper lipid 1,2-dioleoyl-sn-glycero-3-phosphoethanolamine (DOPE) [2,30,31], which effectively delivers NA in vitro and in vivo. We have shown that the incorporation of a folate-containing lipoconjugate (*O*-{2-[*rac*-2,3-di(tetradecyloxy)prop-1-yloxycarbonyl]aminoethyl}-*O*’-[2-(pteroyl-L-glutam-5-yl)aminoethyl]octadecaethyleneglycol) into the lipid composition (liposome F) provides targeted delivery to tumors that express FR at a high level [2,32]. In this study, we examined the in vivo toxicity of liposome F and the antitumor effect of combined antitumor therapy comprising MDR1-targeted siRNA/F complexes and conventional polychemotherapy (PCHT) using tumor xenograft initiated in immunodeficient mice. The novelty of this study is found in the use of original folate-containing cationic liposomes for anti-MDR1-siRNA delivery in conjunction with polychemotherapy, determination of the correlation between the expression of folate receptors in cells and the effectiveness of the use of the studied liposomes as a means of delivering therapeutic siRNAs to these cells.

## 2. Materials and Methods

### 2.1. Liposome Preparation

The cationic lipid 2X3 and the folate lipoconjugate *O*-{2-[*rac*-2,3-di(tetradecyloxy)prop-1-yloxycarbonyl]aminoethyl}-*O’*-[2-(pteroyl-L-glutam-5-yl)aminoethyl]octadecaethyleneglycol (FC) were synthesized as we have described previously [2,30,32]. Briefly, 1,2-di-*O*-ditetradecyl-*rac*-glycerol was activated with 4-nitrophenyl chloroformate and treated with PEG diamine. The obtained product was condensed with FA in the presence of *N,N,N′,N′*-tetramethyl-*O*-(benzotriazol-1-yl) uronium tetrafluoroborate (TBTU) and *N,N*-diisopropylethylamine (DIPEA) to give the desire folate lipocongugate. Liposomal formulations L (2X3/DOPE) and F (2X3/DOPE/FC) were prepared by hydrating thin lipid films. Briefly, a solution of polycationic lipid 2X3 in a mixture of CHCl_3_–CH_3_OH (1:1, *v*/*v*) was added to a solution of DOPE (Avanti Polar Lipids, Alabaster, AL, USA) in CHCl_3_ at a molar ratio of 1:2, and gently stirred. For liposomal formulation F, a solution of FC in CHCl_3_–CH_3_OH (1:1, *v*/*v*) was added to the 2X3/DOPE mixture (1:2 molar ratio) to give 2 mol% of FC in the formulation. Organic solvents were removed in vacuo. The obtained lipid film was dried for 4 h at 0.1 Torr to remove residual organic solvents and was hydrated in deionized water (Milli-Q) at a concentration of 1 mM of polycationic amphiphile 2X3 at 4 °C overnight. The resulting liposomal dispersion was sonicated for 15 min at 70–75 °C in a bath-type sonicator (Bandelin Sonorex Digitec DT 52H, Berlin, Germany), filtered (0.45 μm Chromafil^®^ CA-45/25; Macherey–Nagel, Dueren, Germany), flushed with argon, and stored at 4 °C.

### 2.2. siRNAs

The sense and antisense strands of siRNAs were synthesized, isolated, and characterized as described previously [31]. Here we used the 2′-*O*-methylated siRNA termed siMDR, targeted to nucleotides 411–431 of human *MDR1* mRNA (sense strand 5′-GCGCGAGGUCGGGAUmGGAUCU-3′; antisense strand 5′-AUCCmAUCCCGACCUCGCGCUC-3′; and siScr, with no significant homology to any known mouse, rat, or human mRNA sequences (sense strand 5′-GCUUGAAGUCUUUmAAUUmAAGG-3′; antisense strand 5′-UUmAAUUmAAAGACUUCmAAGCGG-3′), note that 2′-*O*-methyl-modified C and U nucleotides are designated as Cm and Um). A minohexyl group was introduced at the 3′-end of the antisense strand of siMDR using the C6 CPG 3′-PT-amino-modifier (Glen Research, Sterling, VA, USA) during standard solid-phase synthesis. Cyanine7 (Cy7) or Alexa Fluor 488 were attached to siMDR through a 3′-aminohexyl linker according to the manufacturer’s protocol, using Cy7 *N*-hydroxysuccinimide ester (Biotech Industry Ltd., Moscow, Russia) or Alexa Fluor 488 sulfodichlorophenol ester (Thermo Fisher, Waltham, MA, USA) in 0.1 M Tris buffer (pH 8.4). Isolation of the oligoribonucleotides and their conjugates was accomplished by electrophoresis in a 12% denaturing polyacrylamide gel (dPAAG). The purified oligoribonucleotides were characterized by MALDI-TOF-MS. siRNAs (50 μM) were annealed in a buffer containing 30 mM HEPES-KOH (pH 7.4), 100 mM sodium acetate, and 2 mM magnesium acetate, by heating at 90 °C for 5 min, followed by cooling to room temperature. The siRNA preparations were stored at −20 °C until use.

### 2.3. Preparation of the Complexes of Cationic Liposomes and siRNA

For the in vivo studies, the cationic liposomes and siRNA complexes (the ratio of positively-chargeable polymer amine (N = nitrogen) groups to negatively-charged nucleic acid phosphate (P) groups N/P = 1/1) were formed in a volume of 200 µL by mixing equal volumes of liposome L or F (final concentration of 73.5 µM) and siRNA (final concentration of 7 µM) solutions in serum-free OptiMEM (Invitrogen, Waltham, MA, USA). The resulting mixtures were incubated for 20 min at room temperature before administration to mice.

### 2.4. Liposome Sizes and Zeta Potentials

Particle size and zeta potential were measured using a Zetasizer Nano ZS (Malvern Panalytical Ltd., Malvern, UK). For lipoplex characterization, 50 µL of siRNA solution prepared in MilliQ water was mixed with 50 µL of liposome solution taken at the appropriate N/P ratio. Then, after 20 min of incubation at room temperature, the analysis of size was performed using a 100 µL microcuvette. For zeta-potential determination, 900 µL of MilliQ water was added to the sample and surface potential was recorded in a 1 mL cuvette.

### 2.5. Cell Lines and Culture Conditions

Melanoma B16 cells were obtained from the N. N. Blokhin Cancer Research Center, Moscow, RF. Hepatocellular carcinoma G-29, lymphosarcoma LS and RLS, and lung Lewis carcinoma LLC cells were obtained from the Institute of Cytology and Genetics SB RAS, Novosibirsk, RF. Human carcinoma KB-3-1 and mouse leukemia L1210 cells were obtained from the Institute of Cytology RAS, Saint Petersburg, RF. KB-8-5 cells were generously provided by Prof. M. Gottesman (National Institutes of Health, Bethesda, MD, USA).

All cell lines were grown in DMEM (Sigma-Aldrich, St. Louis, MO, USA) supplemented with 10% fetal bovine serum (FBS) (GE Healthcare, Chicago, IL, USA), 100 μg/mL penicillin, 100 μg/mL streptomycin, and 0.25 μg/mL amphotericin, at 37 °C in a humidified atmosphere containing 5% CO_2_/95% air. KB-8-5 cells were cultivated in the presence of 300 nM of vinblastine.

### 2.6. Mice and Tumor Models

All animal procedures were carried out in strict accordance with the recommendations for proper use and care of laboratory animals (ECC Directive 86/609/EEC). The protocol was approved by the Inter-Institute Bioethics Commission of the Siberian Branch of the Russian Academy of Sciences (SB RAS) (#22.11 from 30.05.2014). Eight-to-twelve-week-old male C57Bl/6 mice with an average weight of 16–20 g from the vivarium of the Institute of Cytology and Genetics SB RAS were used to study the acute and chronic toxicity of liposomal formulations. Eight-to-ten-week-old female SCID (SHO-PrkdcscidHrhr) mice with an average weight of 20–22 g from the same source were used to study the biodistribution and antitumor activity of the formulations. The experiments with SCID mice were performed in the Center for Genetic Resources of Laboratory Animals at the Institute of Cytology and Genetics, SB RAS (RFMEFI61914X0005 and RFMEFI62114X0010).

The following cell lines were used as the tumor models: mouse L1210 and LLC, and human KB-3-1 and its multidrug-resistant derivative KB-8-5. Tumors were generated in mice by inoculation of 1.5 × 10^5^ of L1210 or LLC and 1 × 10^6^ of KB-8-5 or KB-3-1 cells in 200 μL of 0.9% saline solution subcutaneously into the right hind legs of SCID mice. Once tumors had reached a palpable volume of approximately 1000 mm^3^ (unless otherwise specified), mice were randomly assigned to experimental or control groups.

### 2.7. Acute and Chronic Toxicity

For assessment of acute toxicity of liposomal formulations, C57BL/6 mice were injected once intravenously with liposome L or F formulations at doses corresponding to 1 (1.5 nmol/g) or 5 (7.5 nmol/g) TD in 200 μL of OptiMEM. Control animals received 200 μL of OptiMEM. The animals were observed for 14 days and then euthanized on day 14 of the experiment. Each experimental group contained six mice.

Mice (*n* = 10) received four IV injections with intervals of 4–5 days at doses corresponding to 1 (1.5 nmol/g) or 2 (3 nmol/g) TD of formulation L in 200 μL of OptiMEM to study chronic toxicity. Control animals received 200 μL of OptiMEM in a similar regimen. The animals were observed for 60 days. Five mice from each group were euthanized on day 21 and all other mice (*n* = 5) from the same group were euthanized on day 60 of the experiment.

During the experiments, the general status of the animals and body weight were monitored. At the end of each experiment or at the intermediate timepoints, organs and peripheral blood were collected to determine organ indexes and complete blood count. The general status of the animals (motor activity, coordination, reaction to stimuli, respiration and heart rate, state of the hair and skin, physiological functions, food and water consumption), body weight, organ indexes (heart, lungs, liver, kidneys, spleen), and complete blood count (leukocytes, lymphocytes, monocytes, granulocytes, erythrocytes, thrombocytes, hemoglobin) were assessed as indicators of toxicity.

Organ indexes were calculated as [organ weight/body weight] × 100%.

Complete blood count was measured on an Auto Hematology Analyzer MicroCC-20Plus (Vet) (High Technology Inc., North Attleborough, MA, USA) with three replicates from each blood sample.

### 2.8. Evaluation of FR Expression by Western Blotting

The level of FR in different mouse tumor cells—lung Lewis carcinoma LLC, hepatocellular carcinoma G29, lymphosarcoma LS and RLS, melanoma B16, and leukemia L1210—and in human KB-3-1 and KB-8-5 carcinomas was analyzed by Western blotting. Cells (3 × 10^5^ cells per sample) were lysed in Laemmli buffer (Sigma-Aldrich), loaded onto a 10% sodium dodecyl sulfate (SDS)/polyacrylamide gel, and then separated at 60 mA for 1 h. The proteins were transferred onto a polyvinylidene difluoride membrane (Millipore, Burlington, MA, USA) using the SemiPhor apparatus (Hoefer, San Francisco, CA, USA), and the membrane was then incubated overnight in 0.5% nonfat dried milk in 0.05 M Tris-HCl, 0.15 M NaCl, 0.1% Tween-20 (pH 7.5) to block nonspecific protein binding. The membranes were incubated for 1 h with monoclonal anti-FR (Abcam, Cambridge, United Kingdom) and anti-β-actin (Sigma-Aldrich) diluted at 1:2000 and 1:5000, respectively. The membranes were washed in phosphate-buffered saline (PBS) supplemented with 0.1% Tween-20 and then incubated for 30 min with secondary goat anti-rabbit antibody conjugated with peroxidase (Abcam). Visualization was performed using ECL Western Blotting Substrate Kit (Abcam) and X-ray film (Carestream, Rochester, NY, USA). The data were analyzed using the GelPro 4.0. software (Media Cybernetics, Rockville, MD, USA). Three independent experiments were performed.

### 2.9. In Vivo Biodistribution Studies

In vivo real-time fluorescence imaging was used to evaluate the distribution of Cy7-labeled siMDR in tumor-bearing mice. The In Vivo MS FX PRO Imaging System (Carestream) was used to obtain X-ray and, concurrently, near-infrared fluorescence (NIRF) images (Cy7: excitation 760 nm, emission 830 nm). Human KB-3-1 or KB-8-5, or mouse L1210 or LLC tumors were generated in SCID mice as described above and were allowed to grow to approximately 1000 mm^3^ in volume. Mice (two mice per group) received an IV injection of 1 µg/g Cy7-siMDR complexed with cationic liposome F (1 nmol/g) at an N/P ratio of 1/1 in 200 μL of OptiMEM medium. The animals were anesthetized with avertin (150 mg/kg, intraperitoneally) and placed on a heating tray (37 °C). The fluorescence (60 sec exposure) and X-ray (60 sec exposure) scans were obtained at different time points (20 min, and 2, 4, 6 and 24 h) post-injection. At the end of the experiment, the mice were euthanized, then the brains, lungs, hearts, livers, spleens, kidneys, and tumors were collected. Each organ was rinsed with PBS, and the fluorescence intensity was detected. Subsequently, the fluorescence intensity from tumors and organs was determined by creating an automatic region of interest (ROI) with a threshold of 30% of each sample’s maximum intensity, measuring that area’s mean by multiplying each luminance level by the number of pixels at that level, and then dividing by the total number of gray levels. Fluorescence intensities were normalized to the peak angle of detection and the area of the organ, and figures were then created in Origin 6.1 (Microsoft Corporation, Redmond, WA, USA). Images were batch exported as 16-bit tiffs, and overlays were completed in Adobe Photoshop CS3 (Adobe, San Jose, CA, USA). Three independent sets of experiments with identical experimental set-ups were performed.

### 2.10. Confocal Microscopy

An experimental scheme similar to that used in whole-body biodistribution studies was used for the microscopic study of Alexa Fluor 488-labeled siMDR accumulation in the organs. Tumors and organs of mice were collected 24 h after injection and immediately frozen with Tissue-Tek O.C.T. (Sakura Finetek Europe, Alphen aan den Rijn, Netherlands) in liquid nitrogen and were kept at −70 °C until processing. Seven micron-thick sections were cut using a Microm HM 505N cryostat (Microm, Germany) at −21 °C. The cryosections were stained with DAPI (4′,6-diamidino-2-phenylindole) and phalloidin-TRITC (tetramethylrhodamine) according to the standard protocol, and mounted in ProLong Gold Antifade Mountant (Life Technologies, Carlsbad, CA, USA). Finally, the cryosections were observed using an LSM 780 confocal fluorescent microscope (Carl Zeiss, Jena, Germany) at 200× magnification using BP 420–480 nm, BP 505–530 nm, and LP 560 nm optical filters.

### 2.11. In Vivo Antitumor Assay

Subcutaneous KB-8-5 tumors were initiated in SCID mice as described above. Once the tumors had reached a palpable volume of at least 50 mm^3^, the mice were randomized into three groups (Control, PCHT, siMDR/F/PCHT, n = 10 per group). On days 9, 16, and 23 after tumor implantation, the siMDR/F/PCHT group received siMDR/F complexes performed in OptiMEM at an N/P ratio of 1/1 and at an siMDR dose of 1 µg/g via tail vein injection. The PCHT and siMDR/F/PCHT groups received PCHT (intraperitoneal injections) at doses corresponding to one-fifth of the median lethal dose (LD_50_: doxorubicin, 4 mg/kg; vincristine, 0.1 mg/kg; cyclophosphamide, 50 mg/kg) at days 12, 19, and 26 after tumor implantation. The tumor size was monitored via caliper measurement. On day 35, the mice were sacrificed, and a macroscopic post-mortem analysis was performed. Gross examination of tumors included evaluation of the size of the tumor node, the presence of a capsule, and the presence of necrosis and hemorrhages. Gross examination of the liver included evaluation of the size, shape, consistency, surface, capsule, and condition on a cut. The tumor and liver from each animal were collected, weighed, and fixed in 10% neutral-buffered formalin for further analysis.

### 2.12. Histology

For histological evaluation, fixed tumor nodes and livers were dehydrated in an ascending series of ethanol and xylols, and then embedded in HISTOMIX paraffin (BioVitrum, Saint-Petersburg, Russia). Paraffin-embedded sections (5 μm) were sliced on a Microm HM 355S microtome (Thermo Fisher Scientific, Waltham, MA, USA) and stained with hematoxylin and eosin, microscopically examined, and scanned. For immunohistochemical studies, the deparaffinized tumor sections were incubated with a caspase-3 specific antibody (ab2302, Abcam) followed by incubation with a secondary horseradish peroxidase-conjugated antibody (Spring Bioscience, San Francisco, CA, USA), exposure to DAB (3, 3′ diaminobenzidine tetrahydrochloride) substrate, and staining with Mayer’s hematoxylin. Images were obtained using an Axiostar Plus microscope equipped with Axiocam MRc5 digital camera (Carl Zeiss, Jena, Germany) at 100×, 200×, and 400× magnifications.

Morphometric analysis of tumor and liver sections was performed by point counting, using a counting grid at a total magnification of 400×. The counting grid had 100 testing points in the studied area equal to 3.2 × 10^6^ μm^2^. Ten to 15 random fields were studied in each specimen. Morphometric analysis of tumors included evaluation of the volume densities (Vv) of the normal tumor tissue, the necrotic tumor tissue, the tumor tissue with an inflammatory infiltrate, and the numeric density (Nv) of mitoses in the tumor tissue and caspase-3-positive cells. Morphometric analysis of the liver included evaluation of the Vv of the normal liver parenchyma, hepatocytes with degenerative and necrotic changes, and the Nv of binuclear hepatocytes reflecting the regeneration capacity of the liver. Morphometric analysis of tumor and liver tissue was performed as described previously [33].

### 2.13. Statistical Analysis

The variables are expressed as the mean ± standard error mean (SEM). The data were analyzed with Student’s *t*-test or one-way analysis of variance (ANOVA). The differences between the values are considered statistically significant when *p* < 0.05.

## 3. Results

We have recently shown that cationic liposome F consisting of the cholesterol-based polycationic amphiphile 2X3 (32.7 molar %), the zwitterionic helper lipid DOPE (65.3 molar %), and the folate-containing lipoconjugate (*O*-{2-[*rac*-2,3-di(tetradecyloxy)prop-1-yloxycarbonyl]aminoethyl}-*O’*-[2-(pteroyl-L-glutam-5-yl)aminoethyl]octadecaethyleneglycol) (2.0 molar %) provides effective targeted delivery of NA into FR-overexpressing tumor cells in vitro and in xenograft tumors in vivo [2,32,34]. In this study, we performed a detailed analysis of acute and chronic toxicity of this liposomal formulation in healthy mice, examined the correlation between the abundance of FR on the cell surface and intratumoral accumulation of NA mediated by liposome F, and analyzed the antitumor effect of PCHT supported by anti-MDR1 siRNA delivered by liposome F on a multidrug-resistant KB-8-5 tumor xenograft in SCID mice. In these experiments, the parent liposome L consisting of lipids 2X3 and DOPE and lacking targeted component were used for comparison. Characteristics of liposomes F and L as well as their complexes with siRNA and their stability in the presence of serum are presented in Table 1 and Figure S1, respectively.

### 3.1. Formulations L and F Have No Acute and Chronic Toxicity in Mice

**Figure 1:**
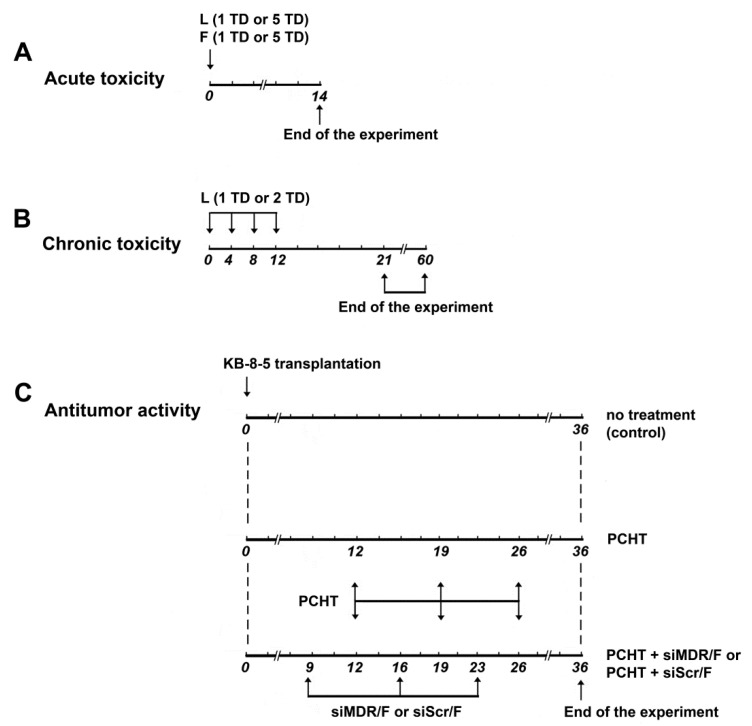
Experimental setup. Analysis of acute (**A**) and chronic (**B**) toxicity of liposomal formulations F and L in healthy C57Bl/6 mice. Note that 1 therapeutic dose (TD) corresponds to 1.5 nmol/g and 5 TD corresponds to 7.5 nmol/g. Analysis of antitumor effects of combination therapy in KB-8-5 tumor-bearing SCID mice (**C**). Combination therapy comprised polychemotherapy (PCHT) with doxorubicin, vincristine, and cyclophosphamide, alongside injection of siMDR/F lipoplexes.

The potential toxic effects of liposomal formulations F and L in vivo were studied using healthy C57BL/6J mice. In this experiment, the general status, body weight, organ indexes, and peripheral blood of experimental animals were analyzed (see Figure 1A,B for the experimental setup). Acute toxicity was examined after a single IV administration of 1 or 5 TD of liposomes F or L (1 TD corresponds to the amount of liposomes used to form lipoplexes with siRNA [1 mg/g] at an N/P ratio of 1/1 and is equal to 1.5 nmol/g); subsequently, the mice were observed for 14 days (Figure 1A). To evaluate chronic toxicity, the mice received four IV injections of 1 or 2 TD of the formulation and were observed for 60 days (Figure 1B).

During the experiments on acute and chronic toxicity, there were no changes in body weight and organ indexes, no animal deaths, and no other manifestations of systemic toxicity for formulations F and L (Table 2 and Table 3). Moreover, there were no differences between the groups injected with either formulation, indicating that incorporation of the folate lipoconjugate in formulation L does not increase the toxicity of the formulation.

On days 21 and 60 after four IV injections of formulation L, a complete blood count in C57BL/6J mice was performed (Table 4). Evaluation of peripheral blood parameters such as leukocytes, lymphocytes, monocytes, granulocytes, and thrombocytes revealed that none of the differences between the groups were significant. Thus, liposomal formulations F and L have no toxic effects in healthy C57BL/6J mice after IV administration, even at long-term observation.

### 3.2. The Abundance of FR in Tumor Cells of Different Origin

**Figure 2:**
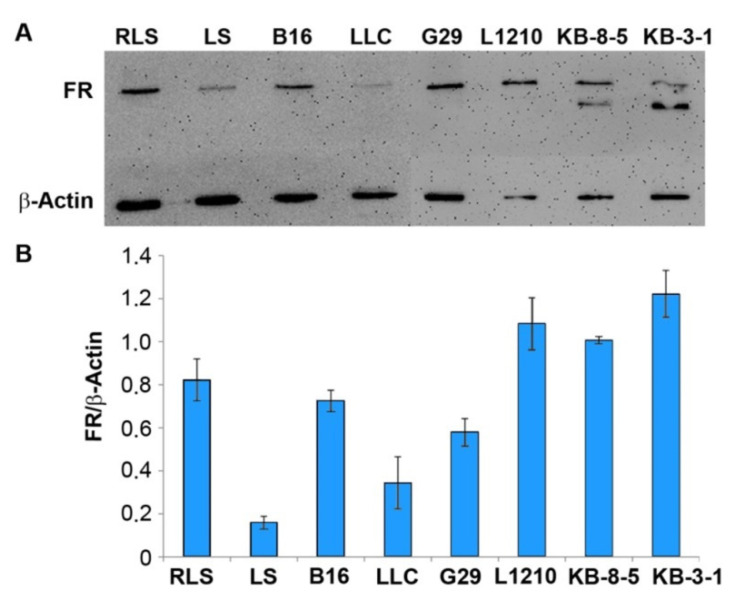
The level of folate receptor (FR) expression in tumor cells measured by Western blot analysis. (**A**) An example of Western blot analysis of the FR level in tumor cell lysates. (**B**) The relative level of FR expression in mouse (lymphosarcoma RLS and LS, melanoma B16, Lewis lung carcinoma LLC, hepatocellular carcinoma G29, and leukemia L1210 cell lines) and human (carcinoma KB-3-1 and its multidrug-resistant analog KB-8-5) tumor cells. The level of β-actin was used as an internal standard. The data represent the mean ± standard error of the mean from three independent experiments.

Liposomal formulation F provides an efficient accumulation of NA in the KB-8-5 tumors that overexpressed FR [17]. We evaluated the abundance of FR in mouse tumor cell lines of different origins by Western blotting to determine how the FR expression levels in target cells affect the delivery efficacy of therapeutic NA in folate-containing liposomes and to determine the range of applicability of these formulations. The following panel of tumor cells able to form a solid tumor in mice was analyzed: Lewis lung carcinoma LLC, hepatocellular carcinoma G29, melanoma B16, leukemia L1210, multidrug-resistant lymphosarcoma RLS, and its parent cell line LS. We also used human carcinoma KB-8-5 cells, with a multidrug-resistant phenotype, and its parent cell line KB-3-1, with high FR expression, for comparison [18]. It should be noted that the antibody we used detects both mouse and human FR. The maximum level of FR was observed in human carcinoma KB-3-1 and KB-8-5, and in mouse leukemia L1210 cells (Figure 2). The hepatocellular carcinoma G29, melanoma B16, and lymphosarcoma RLS cells showed a rather high abundance of FR: The level of FR was only 1.5–2-fold lower than the corresponding level in the control KB-3-1 cells. The lymphosarcoma LS cells had the lowest FR expression: It was 7.5-fold lower than in the KB-3-1 cells. It should be noted that the multidrug-resistant lymphosarcoma RLS cells had a five-fold higher abundance of FR than its parent line LS (Figure 2).

### 3.3. Biodistribution of siMDR in Tumor-Bearing Mice and Confocal Microscopy of Tumor Sections

**Figure 3:**
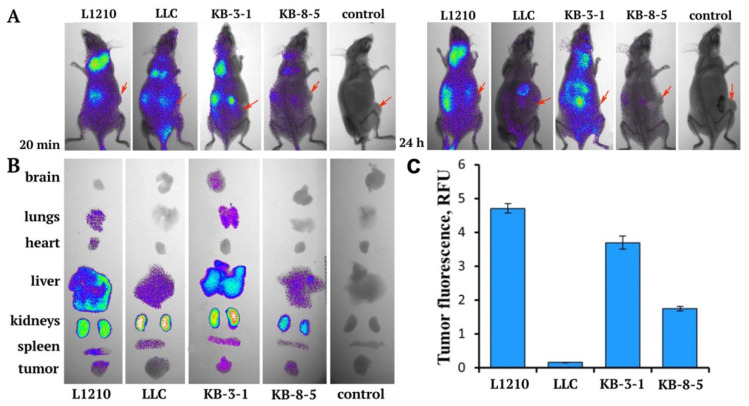
Biodistribution of Cy7- siMDR/F lipoplexes in tumor-bearing SCID mice. (**A**) Images of the whole body of SCID mice at 20 min and 24 h after intravenous (IV) injection of the lipoplexes. Control: non-injected mice. (**B**) Images of dissected organs and tumors of SCID mice at 24 h after IV injection. (**C**) Quantitative data of Cy7-siMDR accumulation in tumors 24 h after IV injection of Cy7-siMDR/F lipoplexes. Arrows indicate tumor localization.

According to the data on the abundance of FR on the surface of various tumor cells revealed by Western blotting, we selected the KB-3-1, KB-8-5, and L1210 cells lines—with high FR expression—and one line (LLC) with low FR expression to analyze the accumulation of siMDR/F lipoplexes in xenograft tumors formed by these cells in SCID mice. Xenograft tumors were initiated in SCID mice by subcutaneous injection of the cells (for details, see Materials and Methods). Immunodeficient mice were selected to implant all tumor cells to avoid possible variations in biodistribution in different mouse strains for syngeneic tumors and xenografts. When the tumor volume reached approximately 1000 mm^3^ in volume, three tumor-bearing mice per group received an IV injection of 10 µg Cy7-siMDR complexed with liposomes F (N/P = 1/1) in 200 µL of OptiMEM; non-injected tumor-bearing mice were used as the control. This ratio was selected on the basis of our previous data, which indicated that at an N/P ratio of 1/1, delivery was predominantly mediated by FR, while an increase in the content of cationic lipids in the complex led to enhanced nonspecific delivery of the cargo [2]. Mice were imaged simultaneously at the indicated time points. Figure 3A shows representative images of mice taken at 20 min and 24 h post-IV injection.

The accumulation of Cy7-siMDR in tumors mediated by formulation F occurred with different efficacy (Figure 3B,C). The most efficacious accumulation of Cy7-siMDR occurred in the xenografts formed by L1210 cells, with an average fluorescence of 4.7 RFU (relative fluorescence units). The efficacy of Cy7-siMDR accumulation in the KB-3-1 xenografts was slightly lower (3.7 RFU), whereas the accumulation in the KB-8-5 xenografts was significantly lower at 1.75 RFU. The accumulation of siMDR in the tumor formed by LLC cells was insignificant (<0.1 RFU) and correlated well with a low level of FR expression in these cells. It should be noted that the tumor type had virtually no effect on the accumulation of Cy7-siMDR/F complexes in the internal organs of mice. Specifically, Cy7-siMDR/F complexes mainly accumulated in the kidneys and liver, with less accumulation in the spleen. Variations in accumulation of the lipoplexes in the lungs and heart of L1210 tumor-bearing mice and in the lungs and brain of KB-3-1 tumor-bearing mice, observed in some animals, indicated insignificant levels of fluorescence and may be explained by differences in blood content of organs from individual animals. Thus, the data revealed that the accumulation of siMDR/F lipoplexes in various types of tumors strongly depends on the abundance of FR in tumor cells, and effective accumulation occurs only in tumors formed by cells with the highest FR levels.

**Figure 4:**
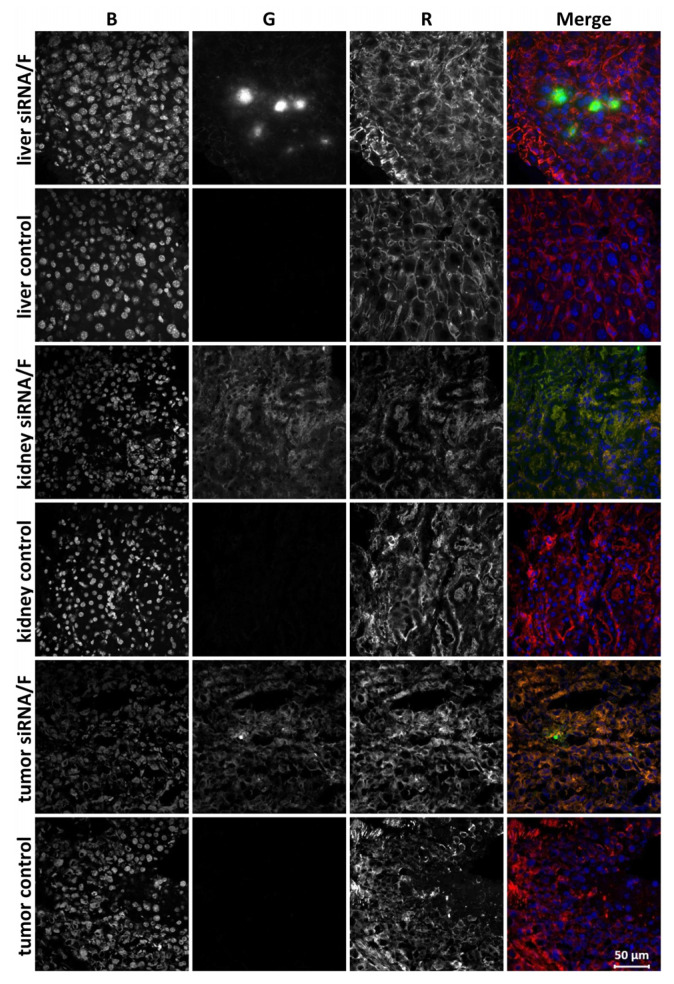
Accumulation of Alexa Fluor 488–siMDR in the tumor and internal organs of KB-3-1 tumor-bearing SCID mice 24 h after intravenous (IV) injection of Alexa Fluor 488–siMDR/F lipoplexes. Localization of Alexa Fluor 488–siMDR was analyzed by confocal fluorescence microscopy at 200× magnification. Three-channel (RGB) pictures were obtained using Alexa Fluor 488 (R), attached to siMDR; actin filaments are stained by TRITC-phalloidin (G) and DNA is stained with DAPI (B).

Cellular localization of siMDR in the tumor and internal organs of tumor-bearing SCID mice was analyzed by confocal microscopy 24 h after IV injection of Alexa Fluor 488–siMDR/F lipoplexes in KB-3-1 tumor-bearing mice. We examined cryosections of the liver, kidneys, and KB-3-1 tumor, because these particular organs had the maximum accumulation of siMDR/F lipoplex at 24 h post-injection. Alexa Fluor 488–siMDR was evenly distributed throughout the tumor volume, a finding that was consistent with the bioimaging data. The tumors appeared as homogeneous round cells. Within the cells, Alexa Fluor 488–siMDR was accumulated in the cytoplasm and was absent in the nuclei or was detected in the nuclei in a small amount (Figure 4). There were minor areas or even individual cells in the sections that contained higher concentrations of Alexa Flour 488–siMDR, but they were not visually distinguished from other areas or neighboring cells by morphology. Generally, the localization of Alexa Fluor 488–siMDR/F within the tumor was rather uniform.

The total fluorescent signal in the kidney cross-sections was more intense than in the tumor sections and, unlike the tumor, the cells with high fluorescence were uniformly distributed throughout the tissue. Similar to the tumor, the kidney cell nuclei lacked Alexa Fluor 488–siMDR. By contrast, siMDR was not distributed uniformly in the liver: There was a significant accumulation of Alexa Fluor 488–siMDR in some regions or individual cells while it was absent in others. Thus, we demonstrated that siMDR delivered within lipoplexes with formulation F spread deep into the tissue and was present in the cytoplasm of almost all cells of the kidneys and the tumor. These organs can be considered as targets for siRNA delivery by formulation F.

### 3.4. The Effects of Combination Therapy including Anti-MDR1 siRNA/F Complexes and PCHT on a Multidrug-Resistant KB-8-5 Tumor Xenograft in SCID Mice

The antitumor effect of combination therapy including the silencing of the *MDR1* gene with siMDR delivered in complex with liposome F followed by PCHT was studied in KB-8-5 human xenograft-bearing SCID mice to assess the therapeutic potential of siRNA-loaded liposomes. KB-8-5 cells are characterized by a high level of P-glycoprotein, which is encoded by the *MDR1* gene, a factor that leads to the resistance of these cells to chemotherapy due to the pumping out of chemotherapy drugs from the cell by P-glycoprotein, with a consequent decrease in their effective concentrations. To restore tumor cell sensitivity to PCHT, we administered siMDR/F complexes 3 days before the PCHT administration (Figure 1). The scheme of treatment is based on our previous data [35] showing that efficient silencing of P-glycoprotein expression (to 40% of the control level) in KB-8-5 tumors occurred 3 days after IV administration of siMDR/F complexes in tumor-bearing SCID mice. In our study, we used PCHT, which is the combination of chemotherapeutic drugs with different mechanisms of action and toxic profiles: cyclophosphamide, doxorubicin, and vincristine. The doses of these chemotherapeutics were selected in accordance with the median lethal doses (LD50), which were measured previously in the experiments on mice for each of the drugs. Mice were injected with the doses corresponding to one-fifth of the LD50 for each of the chemotherapeutic drugs: cyclophosphamide—50 mg/kg, doxorubicin—4 mg/kg, vincristine—0.1 mg/kg, as it was done in our previous works [36,37].

siMDR was targeted to the 411–431 nt region of human *MDR1* mRNA [38]. 2′-*O*-methyl modifications were introduced into nuclease-sensitive sites of siMDR according to the previously developed algorithm [39] to increase the duration of the silencing effect. KB-8-5 cells were subcutaneously injected into SCID mice assigned to one of three experimental groups, and when the tumors reached approximately 700–1000 mm^3^ in volume, the mice were injected with either siMDR/F complexes (at days 9, 16, and 23; siRNA dose was 1 µg/g, N/P = 1/1) and subsequently with PCHT (at days 12, 19, and 26; doxorubicin [4 mg/kg], vincristine [0.1 mg/kg], cyclophosphamide [50 mg/kg]) or with PCHT alone (on the same days) (Figure 1). Untreated tumor-bearing mice were used as the control.

**Figure 5:**
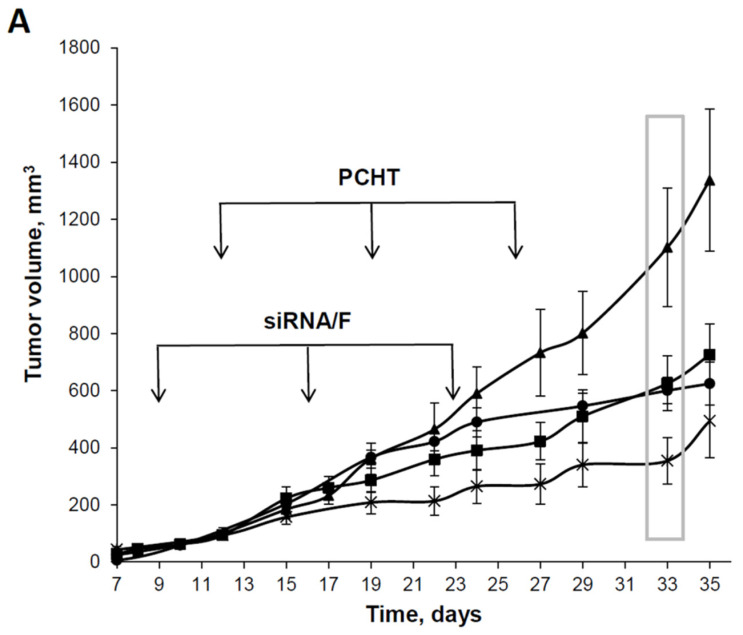

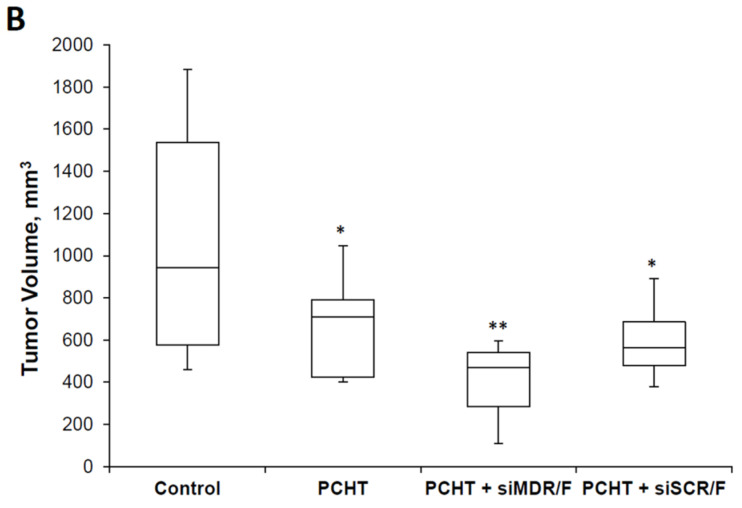
The effect of siMDR/F lipoplexes and polychemotherapy (PCHT) on KB-8-5 human xenograft tumors in SCID mice. The mice received treatment according to the experimental setup (Figure 1). (**A**) Dynamics of tumor growth. The days of siMDR/F or PCHT treatment are shown by arrows. The data represent the mean ± standard error of the mean. PCHT alone (-■-), siMDR/F and PCHT (-×-), siSCR/F and PCHT (-•-), and control, untreated mice (-▲-). (**B**) Tumor volumes on day 33 after tumor implantation (boxed in A). The data were processed using the STATISTICA program, using one-way analysis of variance followed by post hoc analysis (Fisher’s test). The data represent the median, the boxes show the variations between the 25% and 75% percentiles, and the whiskers show the non-outlier range. Statistically significant differences between the control group and other groups (PCHT and PCHT/siMFR/F) are indicated by asterisks (* *p* < 0.05; ** *p* < 0.005).

When examining the dynamics of primary tumor growth, both types of treatment lead to inhibition of tumor growth compared with the control group, but the silencing of *MDR1* with siMDR prior to PCHT led to significantly more effective inhibition of tumor growth compared with PCHT alone (Figure 5A). Thus, on day 33 there was a significant decrease in the tumor size between the control group and both treated groups: siMDR/F/PCHT (*p* < 0.005) and PCHT alone (*p* < 0.05) (Figure 5B). The retardation of tumor growth in mice treated with siMDR/F/PCHT was more pronounced compared with mice treated with PCHT alone. Thereby, mouse tumors formed by drug-resistant cells more efficiently regressed when treated by combination therapy including siMDR/F and PCHT than by PCHT only. These data confirm that formulation F provides an efficient accumulation of siMDR in tumors and ensures the manifestation of its biological activity.

### 3.5. siMDR/F Combined with PCHT Reduces Necrosis and Inflammation, and Stimulates Apoptosis in KB-8-5 Tumor Tissue

The gross examination of KB-8-5 tumors in control mice and mice after treatment revealed necrotic foci in the central part of the tumor nodes. The necrotic decay was more pronounced in the tumors in the control group compared with the PCHT group or the siMDR/F/PCHT group. In all groups, the tumors had a rounded shape and clear boundaries with surrounding tissues.

**Figure 6:**
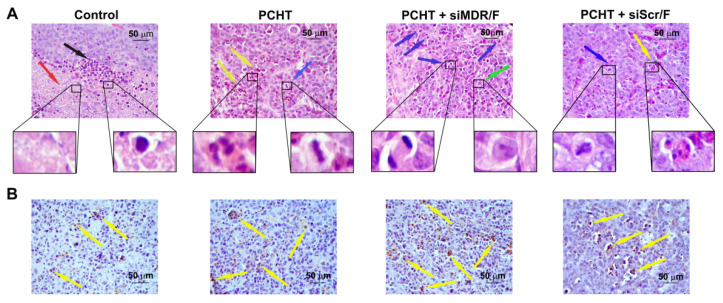
Histopathological analysis of KB-8-5 tumor tissue from SCID mice without treatment (control), after polychemotherapy (PCHT), after PCHT combined with siMDR/F treatment (PCHT + siMDR/F) and after PCHT combined with siScr/F treatment (PCHT + siScr/F). (**A**) Tumor sections stained with hematoxylin and eosin. (**B**) Tumor sections stained with an anti-caspase-3 monoclonal antibody. The infiltration of tumor tissue by inflammatory cells is indicated by the black arrow. Necrotic tumor tissue is indicated by the red arrow. Apoptotic tumor cells are indicated by the yellow arrows. Mitoses are indicated by the blue arrows. Normal tumor cells are indicated by the green arrow. The total magnification is 400×.

Under the microscopic examination, the tumor tissue presented polymorphic epidermal cancer cells with acidophilic cytoplasm, a large nucleus with 2–3 hyperchromic nucleoli, and a high mitotic rate (Figure 6). In the tumors from all groups, there were foci of necrotic decay and inflammatory infiltration (Figure 6). Inflammation was represented by lymphocytes, macrophages, and a small number of neutrophils; it was located at the border of normal tumor tissue and necrosis (Figure 6). However, areas of necrosis and inflammation were much smaller in the siMDR/F/PCHT and PCHT groups compared with the control group. The Vv of necrotic changes and inflammatory infiltration in the tumor tissue of control animals was 33.7 ± 2.1% and 33.0 ± 2.6%, respectively (Table 5). PCHT treatment led to a 1.3-fold decrease in necrosis and did not affect the inflammation in the tumor tissue (Table 5, Figure 6B), while combination therapy (siMDR/F/PCHT) resulted in a significant reduction in pathological changes in tumor tissue: a 2.7-fold decrease in the Vv of necrosis and a 4.5-fold decrease in the Vv of inflammation (Table 5, Figure 6A). This can be associated with the larger size of the tumor node in the control group and with its insufficient trophic and blood supply. Importantly, there was a decline in the Nv of mitoses in the siMDR/F/PCHT group but not the PCHT group; this parameter was unaffected in the latter group (Table 5, Figure 6A).

Morphometric analysis of caspase-3 immunohistochemical images demonstrated that KB-8-5 tumors in the control group had fewer apoptotic cells and the Nv of caspase-3 positive cells was 7.1 ± 1.8 (Figure 6B). PCHT (2.3-fold) and especially siMDR/F/PCHT (3.4-fold) therapy increased the number of caspase-3 positive cells (Figure 6B), showing induction of apoptotic changes in tumor cells. Thus, siMDR/F/PCHT therapy reduces negative morphological changes in tumor tissue, such as necrosis and inflammation and stimulates apoptosis of KB-8-5 cells.

### 3.6. siMDR/F Combined with PCHT Does Not Induce Liver Toxicity in KB-8-5 Tumor-Bearing Mice

The typical liver structure comprises hepatic lobules, which in turn consist of ropes arranged radially around the central vein. As a rule, the liver of healthy animals contains approximately 10% destructive changes (5% of which are dystrophy and 5% of which are necrosis) (Figure S2A). It is known that exposure to toxic factors leads to disruption of the rope structure and the development of dystrophic and necrotic changes in hepatocytes.

According to the histopathological analysis of the liver tissue of untreated KB-8-5 tumor-bearing mice, the tumor had no toxic effect on the liver (Table S1, Figure S2B). PCHT or siMDR/F/PCHT did not increase the level of destructive changes in the liver (Figure S1C,D): In the experimental groups, the percentage of total destructive changes in the liver was 8.3 ± 1.1% and 8.8 ± 1.2%, respectively (Table S1).

The liver regenerative activity was evaluated by calculating the Nv of binuclear hepatocytes. The growth and development of primary tumors in the control group did not influence this parameter (Table S1). PCHT insignificantly reduced the regenerative activity of the liver compared with the control group, while PCHT combined with siMDR/F had no pronounced effect on the regenerative activity of the liver (Table S1).

Summing up the above data, it can be concluded that siMDR/F/PCHT therapy does not cause liver damage: There were no significant differences in destructive changes and regenerative capacity of the liver between control animals and animals that received siMDR/F/PCHT.

## 4. Discussion

The effectiveness of the liposome composition as a carrier system for NA drugs depends mainly on the physicochemical properties of their membranes, the nature of their components, and their size, surface charge, and lipid organization [40]. For in vivo use of liposomes, it is important to provide a particle size of 50–100 nm; it is preferable that their surface has a neutral or slightly negative charge. Because the packaging of NA into neutral liposomes is a difficult task, the development of NA delivery vehicles uses various structures that shield the charge. Modification of the liposome surface to create a protective layer is considered an effective strategy to increase the residence time of the formulation in the bloodstream. Polyethylene glycol (PEG) is most often used for this purpose because PEGylated nanoparticles are stable in the bloodstream and are efficiently delivered to tumor tissue due to the enhanced permeability and retention (EPR) effect. On the other hand, PEGylation impairs a number of properties of lipid nanoparticles that are critical for achieving a therapeutic effect: steric hindrance, which prevents recognition and excretion of liposomes by the RES, and also prevents them from interacting with the cells [41]. To solve this problem, researchers have proposed to use liposomes with addressing groups attached to PEG [42].

We have previously developed an effective liposomal NA delivery system based on the polycationic amphiphile 2X3 and the lipid-helper DOPE [2,30,43] equipped with a folic acid-containing lipoconjugate [2]. DOPE promotes NA escape from endosomes due to its ability to form an inverted hexagonal phase under conditions of endosomal acidification [44,45,46]. It was also found that the use of DOPE in liposomal compositions induces more compact packaging of nucleic acids [44]. PEG800 was used as a linker to design the lipoconjugate with folate. This choice was based on our earlier data on the study of the properties of liposomes obtained on the same platform 2X3/DOPE, which included lipoconjugates with PEG of various lengths: P800, P1500, and P2000 [43]. We have shown that liposomes containing a longer PEG showed increased stability in the bloodstream and an improved ability to provide interferon-inducing and antiviral effects of immunostimulatory RNA, but showed a lower transfection activity in cell culture [43,47]. Therefore, we used PEG linker to generate a folate-containing lipoconjugate to combine the benefits of PEG-protected liposomes with folate-mediated targeted delivery. We demonstrated that the characteristics of the resulting liposomes meet the requirements for lipid delivery systems for in vivo use: Liposomes L and F are small, compact particles in the solution [2], and the formulations were not toxic to cells. Indeed, even a concentration of 80 μM did not reach the half-maximal inhibitory concentration (IC_50_). We have shown that at an N/P ratio of 1/1, the resulting complexes of siRNA with liposomes F having a negative surface charge, direct folate-dependent siRNA transport into FR-expressing tumor cells, and provide for the accumulation and silencing activity of siRNA in an FR-expressing tumor in vivo [2]. In this study, we used these liposomes and their complexes with siRNAs to determine their applicability and safety for in vivo delivery of siRNAs to tumors.

Cationic liposomes, which are actively used for the delivery of therapeutic NA, themselves can have toxic effects on normal tissues and induce an immune response [48]. It has been shown that they can cause toxicity in macrophages and monocytes, induce the secretion of inflammatory cytokines [18,43,47], and also activate the complement system. The nature and extent of these responses can vary depending on the properties of the liposomes, including surface charge, size, and PEGylation [19,23,33]. Therefore, the assessment of liposomes and lipoplexes formed by them with therapeutic NA was the primary task of the study. We showed that IV administration of liposomes F and L to healthy C57BL/6J mice in escalating doses did not cause acute and chronic toxicity, as indicated by the general status of the animals, blood composition, body weight, and the data of the postmortem examination. According to the histopathological study of SCID mice with KB-8-5 xenograft tumors, three IV administrations of liposomes complexed with chemically modified anti-MDR1 siRNA did not have a damaging effect on the organs of animals receiving polychemotherapy. Specifically, it did not increase the volume of destructive changes in the liver caused by the toxic effect of the tumor and did not reduce the regenerative potential of the liver. Because the preparation did not cause apparent toxic effects, both in healthy immunocompetent mice and in immunodeficient mice with a tumor, we drew a preliminary conclusion that the developed delivery system is safe.

We have previously shown that folate liposomes deliver pDNA with different efficiencies to cell types that differ in the expression of FR [2,32,34]. In the present study, we evaluated the applicability of folate liposomes to deliver NA to tumors in vivo, which differ in the level of FR expression. Direct determination of the level of receptors by Western blotting showed that the expression of FR decreased in the order: KB-3-1 > L1210 > KB-8-5 > RLS > B16 > G29 > LLC > LS cells. Evaluation of the accumulation of siRNA/F complexes in the xenograft tumors formed by three cell lines—KB-3-1, KB-8-5, and L1210—with high expression of FR and one cell line (LLC) with a low level of its expression by in vivo imaging techniques demonstrated that intratumoral siRNA accumulation strongly depends on the abundance of FR in tumor cells, and effective accumulation takes place only in tumors formed by cells with the highest FR levels. These data demonstrate the selectivity of the delivery and underscore the range of applicability of the described delivery system for tumors of different origins. We propose that the selectivity is determined by the fact that for complexes formed at a low N/P ratio, nonspecific delivery into cells is extremely ineffective; therefore, in the absence of specific interaction with the receptor or its low level, there is no significant retardation of lipoplexes in the tumor. It should be emphasized that a low N/P ratio, in addition to ensuring the selectivity of lipoplex accumulation in FR-expressing cells, ensures the maintenance of a weakly negative surface charge of liposomes, which, together with a PEG shield, could contribute to the avoidance of lipoplex absorption by phagocytes. Neutral or weakly negative particle charges are among the requirements for delivery systems for in vivo use. However, packaging of NA in neutral or negatively charged liposomes is problematic. To solve this problem, multilayer liposomes with compacting and covering structures and/or liposomes containing components whose charge is dependent on pH have been developed [49]. The use of complexes of NA with cationic liposomes at low N/P ratios can be a simple and effective solution to this problem.

The synthetic chemistry of lipids makes it possible to obtain a variety of lipids for creating pharmaceutical compositions; however, their properties as carriers of NA are hardly predictable and are determined, as a rule, experimentally after directed synthesis or obtaining libraries [50]. To evaluate the therapeutic significance of the efficacy of siRNA accumulation in a tumor, we used an assessment of the therapeutic effect of anti-MDR1 siRNA, which can suppress the expression of P-glycoprotein responsible for tumor resistance to cytostatics [2,35]. We found that folate liposomes allow delivery of siRNA to the tumor in an amount sufficient to inhibit effectively the expression of the target gene and correspondingly increase the effectiveness of tumor growth retardation. siMDR/F combined with PCHT reduced necrosis and inflammation and stimulated apoptosis in KB-8-5 tumors. Despite the fact that this method did not completely reduce the tumor size, the use of such a therapeutic approach makes it possible to restrain its development. Perhaps this is due to an insufficiently long course of therapy (three injections) after the termination of which the tumor growth rate was increased; however, in mice, carrying out longer courses is extremely difficult due to bioethical issues (tumor volume in the control group) and due to damage to the tail vein as a result of numerous injections. Moreover, it should be noted that for unknown reasons, the accumulation of Cy7-siMDR in the KB-8-5 tumor was not as efficient as in KB-3-1 and L1210 tumors, although the bioperformance of siMDR was similar to that observed in cell culture.

In conclusion, folate-containing liposome F mediated extremely efficacious delivery of siRNA to FR-expressing tumors in vivo and with safe and effective action. The use of lipoplexes with a low content of the lipid component makes it possible to reduce the carrier dose necessary to achieve a therapeutically significant effect, while the introduction of a folate lipoconjugate into the liposomes makes it possible to enhance its accumulation in tumor cells expressing FR. We also cannot exclude that the fine-tuning of the lipoplex surface charge is required to enhance the intratumoral accumulation of siRNA/F lipoplexes in each particular tumor.

## Figures and Tables

**Table 1:** Hydrodynamic diameters, polydispersity index and ξ-potentials of lipoplexes formed by siRNA and cationic liposomes.

Cationic Liposomes	N/P	Diameter, nm	Polydispersity Index (PI)	ξ-Potential, mV
L	-	93.5 ± 1.9	0.4 ± 0.09	45.8 ± 1.1
	1/1	152.6 ± 5.3	0.27 ± 0.04	−14.9 ± 1.8
F	-	117.8 ± 8.9	0.5 ± 0.05	21.9 ± 1.5
	1/1	175.2 ± 22.6	0.3 ± 0.04	−3 ± 0.043

**Table 2:** Analysis of the acute toxicity of formulations L and F in C57Bl/6 mice. Organ indexes of mice on day 14 after a single intravenous (IV) administration of formulations L or F or saline (control).

Group	Average Weight, g	Liver Index ^1^	Renal Index ^1^	Spleen Index ^1^
Control	22.4 ± 0.2	5.8 ± 0.2	0.7 ± 0.07	0.5 ± 0.01
L (1 TD ^2^)	23.2 ± 0.5	5.9 ± 0.2	0.8 ± 0.08	0.4 ± 0.01
L (5 TD ^2^)	22.6 ± 0.3	5.8 ± 0.1	0.7 ± 0.04	0.4 ± 0.01
F (1 TD ^2^)	24.0 ± 0.6	4.8 ± 0.3	0.6 ± 0.03	0.3 ± 0.02
F (5 TD ^2^)	25.6 ± 0.6	5.6 ± 0.3	0.7 ± 0.03	0.3 ± 0.02

^1^ Organ indexes were calculated according to the following formula: organ index = [organ weight (g)/body weight (g)] × 100%.; ^2^ TD, therapeutic dose; 1 TD corresponds to 1.5 nmol/g of liposomes.

**Table 3:** Analysis of the chronic toxicity of formulation L in C57Bl/6 mice. Organ indexes of mice on day 60 after four intravenous (IV) administrations of formulation L or saline buffer (control).

Group	Average Weight, g	Liver Index ^1^	Renal Index ^1^	Spleen Index ^1^	Heart Index ^1^	Lung Index ^1^
Control	20.7 ± 0.5	5.5 ± 0.1	0.7 ± 0.04	0.4 ± 0.02	0.5 ± 0.03	1.2 ± 0.06
L (1 TD ^2^)	21.0 ± 0.4	5.3 ± 0.1	0.7 ± 0.02	0.4 ± 0.02	0.6 ± 0.04	1.2 ± 0.1
L (2 TD ^2^)	20.9 ± 0.3	5.1 ± 0.2	0.6 ± 0.03	0.4 ± 0.03	0.6 ± 0.02	1.2 ± 0.04

^1^ Organ indexes were calculated according to the following formula: organ index = [organ weight (g)/body weight (g)] × 100%.; ^2^ TD, therapeutic dose; 1 TD corresponds to 1.5 nmol/g of liposomes.

**Table 4:** Analysis of chronic toxicity of formulation L in C57Bl/6 mice. Complete blood count of animals after four intravenous (IV) administrations of liposomal formulation L or saline buffer.

Blood Parameter	Day 0 ^1^	OptiMEM		L			
				1 TD ^2^		2 TD ^2^	
		Day 21	Day 60	Day 21	Day 60	Day 21	Day 60
Leukocytes × 10^9^/L	2.5 ± 0.2	3.9 ± 0.1	3.0 ± 0.3	3.1 ± 0.2	2.7 ± 0.4	2.6 ± 0.3	2.4 ± 0.2
Lymphocytes × 10^9^/L	2.2 ± 0.2	2.9 ± 0.3	2.5 ± 0.2	2.6 ± 0.2	2.4 ± 0.4	2.2 ± 0.2	1.9 ± 0.2
Monocytes × 10^9^/L	0.2 ± 0.1	0.5 ± 0.1	0.3 ± 0.1	0.3 ± 0.1	0.3 ± 0.1	0.3 ± 0.1	0.3 ± 0.1
Granulocytes × 10^9^/L	0.2 ± 0.1	0.5 ± 0.1	0.2 ± 0.1	0.2 ± 0.1	0.3 ± 0.1	0.2 ± 0.1	0.2 ± 0.1
Erythrocytes × 10^12^/L	9.7 ± 0.3	9.0 ± 0.2	8.6 ± 0.2	9.4 ± 0.2	8.7 ± 0.5	9.0 ± 0.2	8.8 ± 0.2
Thrombocytes × 10^9^/L	514 ± 94.8	833 ± 113	757 ± 45.9	776 ± 43.6	735 ± 74.9	920 ± 39.9	757 ± 52.3
Hemoglobin g/L	128 ± 4.1	107 ± 3.4	107 ± 3.1	113 ± 2.9	105 ± 5.9	111 ± 3.1	115 ± 3.7

^1^ Day 0 indicates the initial levels of blood parameters before treatment; ^2^ TD, therapeutic dose; 1 TD corresponds to 1.5 nmol/g of liposomes.

**Table 5:** Morphometric analysis of epidermoid carcinoma KB-8-5 tumor tissue without treatment (control), after polychemotherapy (PCHT), and after siMDR/F treatment followed by PCHT (PCHT + siMDR/F).

Morphologic Parameters of Tumor	Control	PCHT	PCHT + siMDR/F	PCHT + siScr/F
Vv ^1^ normal tissue, %	33.1 ± 2.9	45.3 ± 3.6 *	77.7 ± 3.4 *^,#^	52.4 ± 3.9 *
Vv necrosis, %	33.7 ± 2.1	26.5 ± 2 *	14.9 ± 2.5 *^,#^	24.9 ± 4.3 *
Vv inflammation, %	33.0 ± 2.6	27.7 ± 3.1	7.3 ± 1.1 *^,#^	22.8 ± 1.8 *
Nv ^2^ mitosis	2.2 ± 0.2	2.2 ± 0.3	1.4 ± 0.2 *^,#^	2.4 ± 0.3
Nv caspase-3 positive cells	7.1 ± 1.8	16.2 ± 3.7 *	23.9 ± 0.5 *^,#^	12.9 ± 1 *

^1^ Vv—the volume density of the studied histological structure represents the volume fraction of tissue occupied by this compartment.; ^2^ Nv—the numeric density of the studied histological structure indicates the number of particles in the unit tissue volume.; * Statistically significant difference relative to KB-8-5 without treatment (control), *p* ≤ 0.05.; ^#^ Statistically significant difference relative to PCHT group, *p* ≤ 0.05.

## Data Availability

Not applicable.

## Supplementary Materials

The following are available online at https://www.mdpi.com/article/10.3390/pharmaceutics13081252/s1, Table S1: Morphometric analysis of liver tissue of KB-8-5 tumor-bearing mice, without treatment (control), after polychemotherapy (PCHT), and after siMDR/F followed by PCHT. Figure S1. Stability of siRNA/liposome complexes in serum-free Opti-MEM and in Opti-MEM with 10% FBS. Figure S2. Morphological state of the liver tissue of healthy mice (A), mice with a KB-8-5 tumor without treatment (control) (B), after polychemotherapy (PCHT) (C), and after siMDR/F (D) or siScr/F (E) followed by PCHT. Hematoxy-lin and eosin staining. The total magnification is 400×.
