# Supplementary material for: Folate-Equipped Cationic Liposomes Deliver Anti-MDR1-siRNA to the Tumor and Increase the Efficiency of Chemotherapy
Source: Pharmaceutics. 2021 Aug 13;13(8):1252. doi: 10.3390/pharmaceutics13081252 (PMC8399439; doi:10.3390/pharmaceutics13081252)
Supplement: Supplementary file 1 [file pharmaceutics-13-01252-s001.zip › pharmaceutics-1289491-supplementary.pdf]

pharmaceutics

Supplementary Materials: Folate-Equipped Cationic Liposomes Deliver Anti-MDR1-siRNA to the Tumor and Increase the Efficiency of Chemotherapy

Daniil V. Gladkikh, Aleksandra V. Sen′kova, Ivan V. Chernikov, Tatyana O. Kabilova, Nelly A. Popova, Valery P. Nikolin, Elena V. Shmendel, Mikhail A. Maslov, Valentin V. Vlassov, Marina A. Zenkova and Elena L. Chernolovskaya

Table S1. Morphometric analysis of liver tissue of KB-8-5 tumor-bearing mice without treatment (control), after polychemotherapy (PCHT), and after siMDR/F or siScr/F followed by PCHT.

Morphologic parameters of liver	Control	PCHT	PCHT + siMDR/F	PCHT + siScr/F
Normal liver parenchyma, Vv[1], %	87.7 ± 0.6	87.8 ± 1.4	88.8 ± 1.3	88.5 ± 1.2
Dystrophy, Vv, %	2.7 ± 0.5	3.3 ± 0.5	3.4 ± 0.4	3.1 ± 0.5
Necrosis, Vv, %	4.1 ± 0.6	5 ± 0.7	5.4 ± 1.1	5.1 ± 0.4
Total destructive changes, Vv, %	6.8 ± 0.8	8.3 ± 1.1	8.8 ± 1.2	8.2 ± 0.6
Binuclear hepatocytes, Nv[2]	5.4 ± 0.8	3.3 ± 0.5*	4.8 ± 1.2	3.9 ± 0.5*

[1]Vv – the volume density of the studied histological structure represents the volume fraction of tissue occupied by this compartment. [2]Nv – the numeric density of the studied histological structure indicates the number of particles in the unit tissue volume. *Statistically significant difference relative to KB-8-5 without treatment, $p \leq 0.05$.

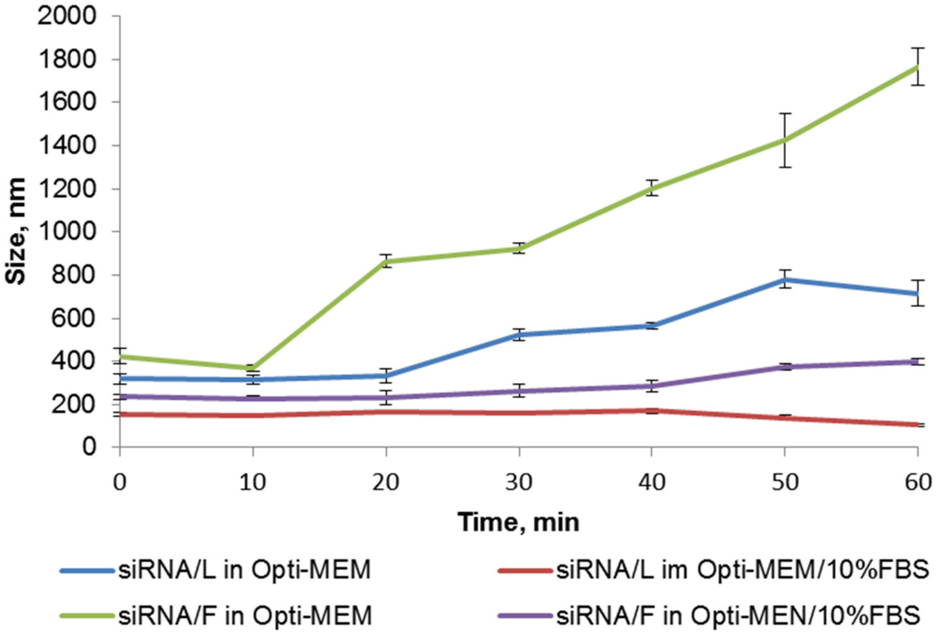

Figure S1. Stability of siRNA/liposome complexes in serum-free Opti-MEM and in Opti-MEM with 10% FBS. Stability of lipoplexes in the presence of serum was evaluated as described in Section 2.4 with modifications: lipoplexes were formed in Opti-MEM medium without serum; then, after 20 min incubation at room temperature, an equal volume of the same medium or medium with 20% FBS was added. The particle size was recorded every 10 minutes for an hour.

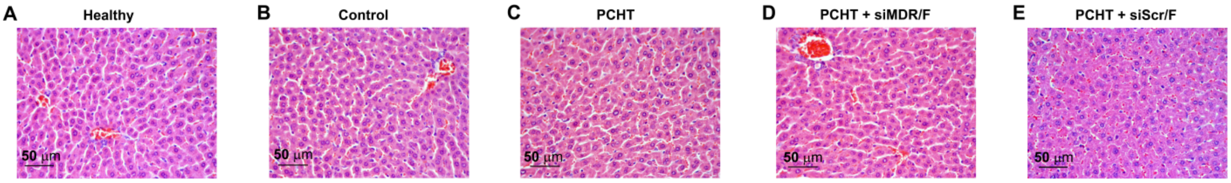

Figure S2. Morphological state of the liver tissue of healthy mice (**A**), mice with a KB-8-5 tumor without treatment (control) (**B**), after polychemotherapy (PCHT) (**C**), and after siMDR/F (**D**) or siScr/F (**E**) followed by PCHT. Hematoxylin and eosin staining. The total magnification is 400×.
